# Work, Diabetes and Obesity: A Seven Year Follow-Up Study among Danish Health Care Workers

**DOI:** 10.1371/journal.pone.0103425

**Published:** 2014-07-28

**Authors:** Kjeld Poulsen, Bryan Cleal, Thomas Clausen, Lars L. Andersen

**Affiliations:** 1 Steno Health Promotion Center, Gentofte, Denmark; 2 National Research Centre for the Working Environment, Copenhagen, Denmark; Old Dominion University, United States of America

## Abstract

**Objectives:**

The rise in prevalence of diabetes is alarming and research ascribes most of the increase to lifestyle. However, little knowledge exists about the influence of occupational factors on the risk for developing diabetes. This study estimates the importance of work and lifestyle as risk factors for developing diabetes mellitus among healthcare workers and explores the association of work factors and obesity, which is a risk factor for diabetes.

**Methods:**

Questionnaire-based prospective cohort study among 7,305 health care workers followed for seven years in the Danish National Diabetes Register. We used bivariate comparisons to give an unadjusted estimate of associations, followed by adjusted survival analysis and logistic regression models to estimate the influences of potential risk factors related to job, health and lifestyle on diabetes and obesity.

**Results:**

During seven years of follow up, 3.5% of participants developed diabetes, associated with obesity (HR  =  6.53; 95% CI 4.68–9.10), overweight (HR  =  2.89; CI 2.11–3.96) age 50–69 y (HR  =  2.27; 95% CI 1.57–3.43) and high quality of leadership (HR  =  1.60; CI 1.19–2.16). Obesity at baseline was most common among the youngest employees, and was mainly associated with developing diabetes (OR  =  3.84; CI 2.85–5.17), impaired physical capacity and physical inactivity. In the occupational setting, obesity was associated with shift work, severe musculoskeletal pain, low influence, but also by good management, fewer role conflicts and a positive work-life balance. Looking only at non-smokers, removed the influence of age and pain. However, non-smokers also had higher depression scores and more role conflicts.

**Conclusions:**

Confirming obesity as the strongest risk factor for developing diabetes, the present study identified few occupational risk factors. However, obesity, the key risk factor for diabetes, had a more variable relation with work than did diabetes.

## Introduction

The rising retirement age in many Western societies means that the workplace will undergo new challenges from increasing numbers of employees with age- and lifestyle-related diseases. However, it is no longer adequate or productive to reduce lifestyle to something only relevant to the private life of individuals because the proliferation of sedentary work in modern employment contributes significantly to the increasing prevalence of lifestyle diseases. One of these is type 2 diabetes mellitus (T2DM), a condition currently reaching epidemic [1], [2] or pandemic [3] proportions, that is, in turn, closely related to the obesity epidemic [3], [4] and the sedentary work and life of many people. Although it is expected that T2DM will influence ability to work [5], most employers are yet not fully aware of, or prepared to meet, this challenge. Furthermore, little knowledge exists about the consequences of T2DM for society, workplaces and employees [6]. It is, however, likely to influence productivity, efficiency, quality and safety [7]–[10], not just because of absenteeism due to sickness, but also because it drains people of both physical and mental energy [11], [12]. In addition, the consequences are greater among the least educated and the socially most vulnerable groups [13]. This is the same category of employees already at higher risk of long term absenteeism, early retirement, disability pension and unemployment [14].

It is estimated that types 1 and 2 diabetes collectively cause the loss of an average of 9 years of life at age 30 years and 3 years at age 70: the relative risk for cardiovascular disease among those with diabetes, compared to those without it, is 3.5 at age 50 [15]. We are unable to distinguish between type 1 and type 2 diabetes in terms of these effects. However, since T2DM accounts for more than 90% of diagnosed diabetes and is the only form of the disease influenced by external factors like lifestyle and work, we refer to T2DM hereafter, unless otherwise specified. Due to the combination of increased prevalence and rising age at retirement, T2DM might soon become one of the most important factors related to the health of the senior workforce. It is surprising, therefore, that so little is known about relationships between T2DM and work.

Since health care workers experience high psychosocial strain and are at increased risk of sickness absenteeism, early labour market exit, musculoskeletal problems and unhealthy lifestyle [16]–[20], they may also be at increased risk of developing diabetes. The prevalence of overweight and physical inactivity is high among health care workers, and the frequent occurrence of physical pain and physical and mental exhaustion from work could reduce the capacity to remain fit and active during leisure. There are also some suggestions that psychosocial strain—especially low control—increases the risk of T2DM [21]–[23], metabolic syndrome [24] and dyslipidemia [25] among women.

The aim of this study is to investigate the potential influence of occupational factors on developing diabetes among healthcare workers. Furthermore, because obesity is the single most important risk factor for T2DM, a second aim was to investigate associations between work and lifestyle factors related to obesity.

## Materials and Methods

Our prospective observational design combined data on lifestyle and working conditions with the risk of developing diabetes by linking a large cohort of female health care workers with the Danish National Diabetes Register (DNDR) [26]. We used 2005 as the baseline year and excluded patients with diagnosed diabetes in 2005 to examine incidence after baseline.

### The health care worker cohort

**Figure 1:**
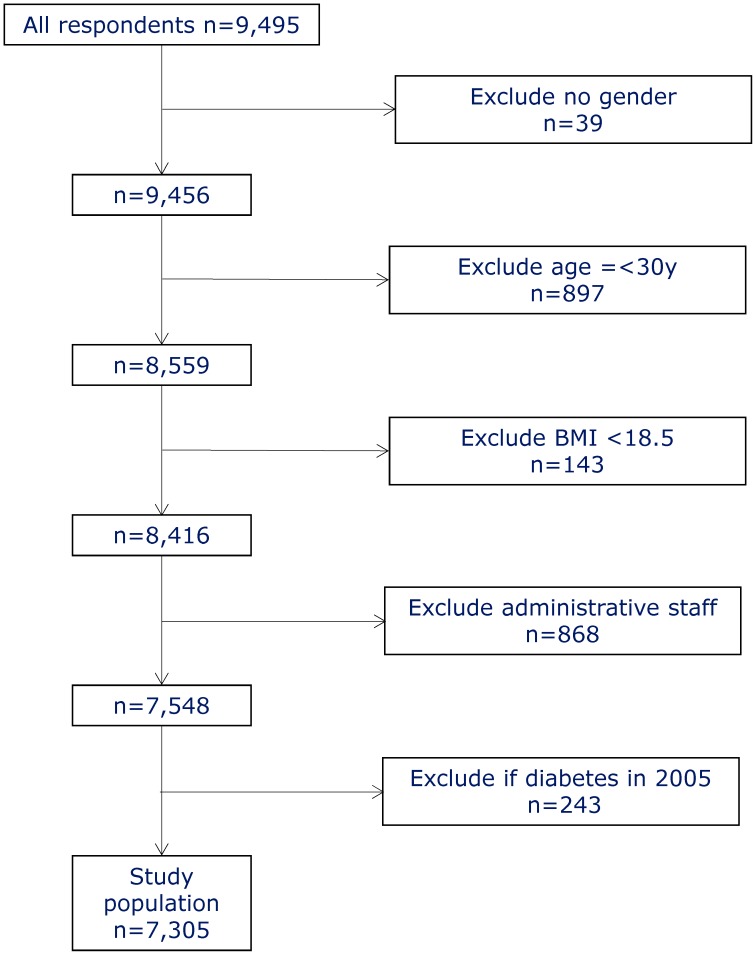
Participant Flow Chart.

The cohort is based on a survey of eldercare service employees conducted in 2004–5 in 35 Danish municipalities. All employees (12,744) received a written questionnaire that was administered by a local contact person in each municipality; 9,495 responded, a response rate of 75%. We excluded respondents who didn't report their gender, underweight workers, and administrative staff. We also excluded workers aged 30 years or younger because of the greater risk of type 1 diabetes (Figure 1). The remaining 7,305 health care workers were linked to disease and population registers via a unique personal identification number and followed for seven years. Detailed information on most of the measures has been reported elsewhere [18]–[20].

### The National Diabetes Register

In 2006 Denmark established a National Diabetes Register (DNDR) that includes all incidences of diabetes since 1995 [26]. DNDR combines data from three different national registries: the National Patient Register (NPR) [27], the National Health Insurance Service Registry (NHISR) [28] and the Register of Medicinal Product Statistics (RMPS) [29]. Individuals must meet at least one of six criteria to be registered in DNDR: 1) receive a diagnosis of diabetes (data source, NPR); 2) receive diabetes-related chiropody care (NHISR); 3) have blood glucose measured five times in a calendar year (NHISR); 4) have at least two annual blood glucose measurements over a period of five consecutive years (NHISR); 5) purchase prescribed oral anti-diabetic medication (registered at second purchase, RMPS); 6) purchase prescribed insulin (RMPS). In addition to the unique personal identification number of each individual, DNDR contains data on sex, date of birth, date of death, and date of inclusion according to the entry criteria. However, the register does not distinguish between type 1 and type 2 diabetes. DNDR has been validated and found to be reliable as a source for linkable information on diabetes at a national level [15], [30].

### Demographic variables

Age, information about ethnicity and household type was drawn from Statistics Denmark [31] through linking with the Danish Civil Registration System [32].

### Measures of health

All measures of health originated from the baseline questionnaires, with the exception of diabetes data from DNDR. General health was self-assessed on a five-point scale ranging from ‘poor’ to ‘outstanding’ and dichotomised as poor if respondents selected one of the two lowest scores and good otherwise. Sleep problems were identified if at least one of five questions was scored higher than three on a six-point scale. Participants separately rated pain in the low back, neck/shoulders and knees as average pain during the last three months on a numerical rating scale from 0–9, where 0 represented ‘no pain’ and 9 represents ‘worst imaginable pain’. Based on the distribution of the rating, pain was categorised in four groups to make it easier to interpret than if using the continuous scale [33]. Depression was evaluated using the Danish version of the Major Depression Inventory (MDI), and the WHO-5 well-being scale was used to assess the risk for developing depression and stress [20], [34].

### Measures of job-related factors

Working hours were self-reported in a single item. Ergonomic strain was assessed with the Hollmann index, a compound measure of body postures and weight lifted during the work day [20]. Strain was stratified in four categories based on the distribution of the continuous compound measure scale. Work-life balance was defined as poor if respondents reported that work was draining both time and energy from their private lives. Assessment of psychosocial working conditions used the same model as in a long-term sickness absenteeism survey [19], in which four scales—emotional demands, influence, role conflict and quality of leadership—were based on four items from the Copenhagen Psychosocial Questionnaire (COPSOQ) [35].

### Measures of lifestyle

All lifestyle variables were self-assessed in the questionnaire. Leisure time physical activity was classified as good if respondents reported they were very active or fairly active. Physical capacity was rated across the domains of physical fitness, strength, endurance and body movement as the average score on a nine-point scale from worst (1) to best (9). On the basis of the distribution of the scores, the authors decided to stratify into four categories. Smokers were those reporting daily smoking. Finally Body Mass Index (BMI) was categorised as normal weight (18.5≤BMI<25), overweight (25≤BMI<30) and obese (BMI≥30).

The study was approved by the Danish Data Protection Agency. In Denmark, however, approval from Ethics Committees is not required for survey research. Furthermore, participation in the study was voluntary which implies that written informed consent is not necessary as this consent is implied in individual respondents' voluntary participation.

### Statistical analyses

Initially, unadjusted bivariate (2×n) analyses compared each variable with the incidence of diabetes using Chi square analysis for trend. All variables were then included in a saturated Cox regression model with diabetes as the dependent variable using SAS Proc Genmod; this was used as the basis for a subsequent manual backward elimination based on log likelihood ratio tests to correct for multiple testing. A similar analytic approach followed, in which obesity at baseline was the dependent variable. However, since this was a cross-sectional design, the Logistic procedure in SAS 9.3 was used for a multiple logistic regression analysis. Manual backwards elimination using the log likelihood ratio test was used to fit the model. The Hosmer and Lemeshow goodness-of-fit test was used to evaluate the logistic analyses [36].

## Results

Health care workers who did not respond to the questionnaire were significantly younger than those who did (OR  =  2.52; 95% CI 2.29–2.75) and more likely to belong to an ethnic minority (OR  =  2.02; 95% CI 1.66–2.43). Non-respondents had a lower incidence of diabetes (OR 0.64 95% CI: 0.48–0.86) though as the fact that they were, on average, younger than respondents provides a potential explanation for this.

Table 1 presents descriptive statistics and the distribution of diabetes incidence. BMI is the single most important risk factor for the development of diabetes, followed by age and lack of physical exercise. Poor general health, musculoskeletal pain and sleep problems predict an increasing diabetes risk, but none of the psychological variables showed significance. From the occupational arena, only shift work (evening/night) and quality of leadership were associated with an increased risk of diabetes.

**Table 1:** Incidence of diabetes among health care workers over seven years.

		N	Developed diabetes	
Demography	Female	7,119	245	(3.6%)
	Male	186	8	(4.3%) p = 0.560
	Age in 2005: 30–39 y	1,475	34	(2.3%)
	Age in 2005: 40–49 y	2,721	68	(2.5%)
	Age in 2005: 50–69 y	3,109	160	(5.2%) **p<0.0001**
	Danish origin	6,977	250	(3.6%)
	Ethnic minority	293	10	(3.4%) p = 0.878
	Live with a partner	6,022	215	(3.6%)
	Live alone	1,283	47	(3.7%) p = 0.871
Health	Good self-rated health	6,398	213	(3.3%)
	Poor self-rated health	862	45	(5.2%) **p = 0.005**
	No musculoskeletal pain	1,115	37	(3.3%)
	Low musculoskeletal pain	2,644	79	(3.0%)
	Moderate musculoskeletal pain	2,821	106	(3.8%)
	Severe musculoskeletal pain	435	28	(6.6%) **p = 0.004**
	No depression	7,161	255	(3.6%)
	Depression	144	7	(4.9%) p = 0.406
	Not at risk for stress or depression	5,962	203	(3.4%)
	At risk for stress or depression	1,343	59	(4.4%) p = 0.079
	No problems with sleep	6,026	202	(3.4%)
	At least one problem with sleep	1,279	60	(4.7%) **p = 0.019**
Work	Work entirely/partly during daytime	5,480	176	(3.2%)
	Work only evening/nights	1,812	86	(4.8%) **p = 0.002**
	Work does not drain time and energy	5,954	210	(3.5%)
	Work drains time and energy	1,264	45	(3.6%) p = 0.946
	Lowest ergonomic strain	2,071	81	(3.9%)
	Low ergonomic strain	1,703	52	(3.1%)
	High ergonomic strain	1,745	54	(3.1%)
	Highest ergonomic strain	1,786	75	(4.2%) p = 0.158
	Low emotional demands	1,319	54	(4.1%)
	Medium emotional demands	4,670	157	(3.4%)
	High emotional demands	1,316	51	(3.9%) p = 0.352
	Low influence	1,588	68	(4.3%)
	Medium influence	3,846	129	(3.4%)
	High influence	1,871	65	(3.5%) p = 0.236
	Low role conflicts	1,449	50	(3.5%)
	Medium role conflicts	4,346	148	(3.4%)
	High role conflicts	1,510	64	(4.2%) p = 0.301
	Low quality of leadership	1,780	73	(4.1%)
	Medium quality of leadership	3,668	100	(2.7%)
	High quality of leadership	1,857	89	(4.8%) **p = 0.0002**
Lifestyle	Exercise	3,898	117	(3.0%)
	No exercise	3,327	138	(4.2%) **p<0.012**
	Lowest self-reported physical capacity	2,633	107	(4.1%)
	Low self-reported physical capacity	1,374	62	(4.5%)
	High self-reported physical capacity	1,493	50	(3.4%)
	Highest self-reported physical capacity	1,805	43	(2.4%) **p = 0.005**
	Non-smoker	4,590	161	(3.5%)
	Smoker	2,672	100	(3.7%) p = 0.604
	Normal BMI	4,308	67	(1.6%)
	Overweight	2,181	107	(4.9%)
	Obese	809	86	(10.6%) **p<0.0001**

Controlling for covariates in the saturated Cox regression model removed most occupational and all health-related variables (Table 2). The risk of developing diabetes was associated with obesity, being overweight, increased age and quality of leadership. These four variables remained significant in the fitted model.

**Table 2:** Survival analysis for 7-year diabetes incidence among health care workers (n = 6,784).

		Full Model		Fitted model^a^	
Demography	Gender (female v male)	0.87	(0.41–1.87)	**-**	**-**
	Age in 2005: (40–49 y v 30–39 y)	1.11	(0.72–1.72)	**-**	**-**
	Age in 2005: (50–69 y v 30–39 y)	**2.37**	**(1.53–3.39)**	**2.27**	**(1.57–3.43)**
	Ethnicity (minority v original Danish)	0.94	(0.46–1.92)	**-**	**-**
	Family (living alone v living with partner)	0.99	(0.70–1.39)	**-**	**-**
Work	Shift work (evening/night v day)	1.27	(0.95–1.70)	**-**	**-**
	Ergonomic strain (low v lowest)	0.81	(0.56–1.17)	**-**	**-**
	Ergonomic strain (high v lowest)	0.82	(0.57–1.19)	**-**	**-**
	Ergonomic strain (highest v lowest)	0.92	(0.63–1.33)	**-**	**-**
	Work-life balance (poor v good)	0.95	(0.66–1.38)	**-**	**-**
	Emotional demands (low v medium)	1.22	(0.86–1.71)	**-**	**-**
	Emotional demands (high v medium)	1.07	(0.76–1.51)	**-**	**-**
	Influence (low v medium)	1.05	(0.75–1.45)	**-**	**-**
	Influence (high v medium)	1.04	(0.76–1.44)	**-**	**-**
	Role conflicts (low v medium)	0.82	(0.57–1.16)	**-**	**-**
	Role conflicts (high v medium)	1.04	(0.75–1.46)	**-**	**-**
	Quality of leadership (low v medium)	1.15	(0.82–1.61)	**-**	**-**
	Quality of leadership (high v medium)	**1.71**	**(1.25–2.32)**	**1.60**	**(1.19–2.16)**
Health	General health (poor v good)	1.11	(0.75–1.64)	**-**	**-**
	Musculoskeletal pain (low v none)	1.05	(0.69–1.60)	**-**	**-**
	Musculoskeletal pain (moderate v none)	1.21	(0.80–1.84)	**-**	**-**
	Musculoskeletal pain (severe v none)	1.49	(0.84–2.65)	-	-
	Sleep (problems v no problems)	1.14	(0.80–1.62)	**-**	**-**
	MDI score (depression v no depression)	0.94	(0.40–2.23)	**-**	**-**
	WHO well-being score (at risk v not at risk)	1.11	(0.78–1.60)	**-**	**-**
Lifestyle	BMI (overweight v normal weight)	**2.84**	**(2.06–3.90)**	**2.89**	**(2.11–3.96)**
	BMI (obese v normal weight)	**6.30**	**(4.27–8.49)**	**6.53**	**(4.68–9.10)**
	Physical exercise (no exercise v exercise)	1.10	(0.83–1.44)	**-**	**-**
	Physical capacity (high v highest)	1.25	(0.82–1.91)	**-**	**-**
	Physical capacity (low v highest)	1.42	(0.94–2.16)	**-**	**-**
	Physical capacity (lowest v highest)	1.11	(0.75–1.67)	**-**	**-**
	Smoking (smoker v non-smoker)	1.21	(0.93–1.59)	**-**	**-**

a Backwards elimination of class variables using log likelihood ratio tests.

Looking at body weight, the youngest employees and those who were ethnically Danish had the highest prevalence of obesity (Table 3). Both physical and mental health problems were associated with obesity. The risk of developing diabetes had the highest odds ratio of all variables in terms of obesity. Shift work and having low influence and scoring high on role conflicts were associated with obesity. Leisure time physical inactivity was associated with obesity, and it is notable that a significantly lower proportion of obese employees were smokers.

**Table 3:** Obesity among health care workers.

		N	BMI≥30	
Demography	Female	7,119	792	(11.1%)
	Male	186	17	(9.4%) p = 0.394
	Age in 2005: 30–39 y	1,475	193	(13.1%)
	Age in 2005: 40–49 y	2,721	289	(10.6%)
	Age in 2005: 50–69 y	3,109	327	(10.5%) **p<0.022**
	Danish origin	6,977	784	(11.2%)
	Ethnic minority	293	22	(7.5%) **p = 0.046**
	Live with a partner	6,022	679	(11.3%)
	Live alone	1,283	130	(10.1%) p = 0.236
Health	Good self-rated health	6,398	668	(10.4%)
	Poor self-rated health	862	140	(16.2%) **p<0.0001**
	No musculoskeletal pain	1,115	106	(9.5%)
	Low musculoskeletal pain	2,644	248	(9.4%)
	Moderate musculoskeletal pain	2,821	347	(12.3%)
	Severe musculoskeletal pain	435	66	(15.2%) **p = 0.0001**
	No depression	7,161	784	(11.0%)
	Depression	144	25	(17.4%) **p = 0.015**
	Not at risk for stress or depression	5,962	641	(10.8%)
	At risk for stress or depression	1,343	168	(12.5%) p = 0.064
	No problems with sleep	6,026	626	(10.4%)
	At least one problem with sleep	1,279	183	(14.3%) **p<0.0001**
	Did not develop diabetes	7,043	723	(10.3%)
	Developed diabetes	262	86	(32.8%) **p<0.0001**
Work	Work entirely/partly during daytime	5,480	566	(10.3%)
	Work only evening/nights	1,812	242	(13.4%) **p = 0.0004**
	Work does not drain time and energy	5,964	673	(11.3%)
	Work drains time and energy	1,264	125	(9.9%) p = 0.151
	Lowest ergonomic strain	2,071	215	(10.4%)
	Low ergonomic strain	1,703	189	(11.1%)
	High ergonomic strain	1,745	181	(10.4%)
	Highest ergonomic strain	1,786	224	(12.5%) p = 0.122
	Low emotional demands	1,319	145	(11.0%)
	Medium emotional demands	4,670	511	(10.9%)
	High emotional demands	1,316	153	(11.6%) p = 0.779
	Low influence	1,588	216	(13.6%)
	Medium influence	3,643	401	(10.4%)
	High influence	1,871	192	(10.3%) **p = 0.001**
	Low role conflicts	1,449	135	(9.3%)
	Medium role conflicts	4,346	485	(11.2%)
	High role conflicts	1,510	189	(12.5%) **p = 0.021**
	Low quality of leadership	1,780	200	(11.2%)
	Medium quality of leadership	3,668	381	(10.4%)
	High quality of leadership	1,857	228	(12.3%) p = 0.103
Lifestyle	Exercise	3,898	320	(8.2%)
	No exercise	3,327	477	(14.3%) **p<0.0001**
	Lowest self-reported physical capacity	2,633	388	(14.7%)
	Low self-reported physical capacity	1,374	178	(13.0%)
	High self-reported physical capacity	1,493	144	(9.6%)
	Highest self-reported physical capacity	1,805	99	(5.5%) **p<0.0001**
	Non-smoker	4,590	570	(12.4%)
	Smoker	2,672	236	(8.8%) **p<0.0001**

Table 4 shows that controlling for confounding removed the association with general and mental health status and role conflicts and added influence from quality of leadership and work-life balance. Apart from introducing the importance of musculoskeletal pain, the fitted regression showed the same pattern as the full adjusted logistic regression model. As smoking is known to reduce obesity, reanalysing the obesity data for non-smokers, we found that smoking could explain the effects of age and musculoskeletal pain. On the other hand, statistical associations of high depression scores and role conflicts with obesity are seen for non-smokers.

**Table 4:** Logistic regression models for obesity among all health care workers (n = 6,784) and only the non-smokers (n = 4.282).

		All health care workers				Only non-smoking health care workers			
		Full Model^a^		Fitted model^b^		Full model^c^		Fitted model^d^	
Demography	Gender (female v male)	1.27	(0.72–2.23)	-	-	1.27	(0.65–2.49)	-	-
	Age (40–49 y v 30–39 y)	**0.76**	**(0.61–0.93)**	**0.76**	**(0.62–0.94)**	0.85	(0.66–1.09)	-	-
	Age (50–69 y v 30–39 y)	**0.67**	**(0.54–0.82)**	**0.68**	**(0.55–0.84)**	**0.76**	**(0.59–0.98)**	-	-
	Ethnicity (minority v original Danish)	**0.54**	**(0.33–0.90)**	**0.54**	**(0.33–0.88)**	**0.45**	**(0.24–0.84)**	**0.45**	**(0.25–0.87)**
	Family (living alone v living with partner)	0.95	(0.77–1.18)	-	-	**1.00**	**(0.77–1.31)**	**-**	**-**
Work	Shift work (evening/night v day)	**1.50**	**(1.25–1.80)**	**1.51**	**(1.26–1.81)**	**1.52**	**(1.21–1.92)**	**1.50**	**(1.25–1.92)**
	Ergonomic strain(low v lowest)	1.05	(0.83–1.31)	-	-	0.99	(0.76–1.30)	-	-
	Ergonomic strain (high v lowest)	0.94	(0.75–1.18)	-	-	1.05	(0.80–1.37)	-	-
	Ergonomic strain (highest v lowest)	1.11	(0.88–1.39)	-	-	1.11	(0.84–1.47)	-	-
	Work-life balance (poor v good)	**0.73**	**(0.58–0.92)**	**0.75**	**(0.60–0.94)**	**0.66**	**(0.50–0.88)**	**0.68**	**(0.51–0.89)**
	Emotional demands (low v medium)	1.04	(0.83–1.30)	-	-	1.19	(0.91–1.55)	-	-
	Emotional demands (high v medium)	1.02	(0.82–1.27)	-	-	1.10	(0.85–1.42)	-	-
	Influence (low v medium)	**1.31**	**(1.08–1.60)**	**1.33**	**(1.10–1.62)**	**1.32**	**(1.04–1.67)**	**1.35**	**(1.07–1.71)**
	Influence (high v medium)	1.04	(0.85–1.27)	-	-	1.05	(0.82–1.34)	-	-
	Role conflicts (low v medium)	0.81	(0.65–1.02)	0.82	(0.65–1.01)	0.85	(0.65–1.11)	-	-
	Role conflicts (high v medium)	1.12	(0.91–1.37)	-	-	**1.33**	**(1.04–1.69)**	**1.40**	**(1.12–1.77)**
	Quality of leadership (low v medium)	0.96	(0.78–1.17)	-	-	0.95	(0.74–1.22)	-	-
	Quality of leadership (high v medium)	**1.28**	**(1.05–1.55)**	**1.29**	**(1.06–1.56)**	**1.35**	**(1.07–1.70)**	**1.32**	**(1.03–1.57)**
Health	General health (poor v good)	1.19	(0.93–1.52)	-	-	1.26	(0.94–1.69)	-	-
	Musculoskeletal pain (low v none)	0.94	(0.73–1.21)	-	-	0.99	(0.73–1.35)	-	-
	Musculoskeletal pain (moderate v none)	1.17	(0.91–1.50)	-	-	1.17	(0.86–1.60)	-	-
	Musculoskeletal pain (severe v none)	1.42	(0.97–2.06)	**1.60**	**(1.12–2.28)**	1.33	(0.83–2.12)	-	-
	Sleep (problems v no problems)	1.22	(0.97–1.52)	-	-	1.10	(0.84–1.45)	-	-
	MDI score (depression v no depression)	1.14	(0.67–1.93)	-	-	1.73	(0.95–3.15)	**2.02**	**(1.11–3.35)**
	WHO well-being score (at risk v not at risk)	0.90	(0.71–1.12)	-	-	0.87	(0.66–1.16)	-	-
	Developed diabetes (yes v no)	**3.80**	**(2.82–5.13)**	**3.84**	**(2.85–5.17)**	**3.30**	**(2.27–4.81)**	**3.32**	**(2.30–484)**
Lifestyle	Physical exercise (no exercise v exercise)	**1.61**	**(1.36–1.91)**	**1.62**	**(1.37–1.91)**	**1.69**	**(1.38–2.07)**	**1.70**	**(1.37–2.05)**
	Physical capacity (high v highest)	**1.68**	**(1.27–2.24)**	**1.69**	**(1.27–2.25)**	**1.78**	**(1.25–2.53)**	**1.81**	**(2.01–3.79)**
	Physical capacity (low v highest)	**2.25**	**(1.70–2.97)**	**2.26**	**(1.71–2.98)**	**2.28**	**(1.61–3.22)**	**2.34**	**(1.68–3.32)**
	Physical capacity (lowest v highest)	**2.31**	**(1.79–3.00)**	**2.37**	**(1.83–3.06)**	**2.66**	**(1.93–3.67)**	**2.81**	**(1.28–2.57)**
	Smoking (smoker v non-smoker)	**0.61**	**(0.51–0.72)**	**0.61**	**(0.51–0.73)**	-	-	-	-

a Hosmer and Lemeshow Goodness-of-fit test: p = 0.9407.

b Log likelihood ratio test used for manual backward elimination. Hosmer and Lemeshow Goodness-of-fit test: p = 0.4955.

c Hosmer and Lemeshow Goodness-of-fit test: p = 0.7268.

d Log likelihood ratio test used for manual backward elimination. Hosmer and Lemeshow Goodness-of-fit test: p = 0.7577.

## Discussion

The present study showed both lifestyle and occupational risk factors among healthcare workers for developing diabetes over a period of seven years. The most important risk factor was a BMI above the normal category. At baseline, obesity was associated with low level of exercise, poor self-rated physical fitness, shift work and problems with health and sleep.

### Diabetes and work

Pain was significantly associated with an increased diabetes risk in the bivariate analysis. In the same cohort, it has been shown that the more pain health care workers have, the higher the long-term sickness absenteeism [33], [37]. After controlling for lifestyle, however, we found little impact from occupational risk factors for developing diabetes. Therefore, if health care workers have so much musculoskeletal pain or are so drained of energy by their work that they cannot stay fit, it will have significant indirect effects on diabetes. The same factors are also expected to cause selection out of work.

Shift work was another factor that increased the risk of developing diabetes in our study. Potential explanations for this finding include the difficulty of maintaining a healthy lifestyle, e.g., a tendency to eat unhealthy foods and exercise less, and effects on the endocrine circadian rhythm [38]–[40]. Although a review of the epidemiological evidence found only moderate support for an association between shift work and metabolic syndrome and limited evidence for a relationship to diabetes [41], those with shift work who already had diabetes experienced more problems maintaining glycaemic control [42].

Interestingly, we found that quality of leadership influenced diabetes risk, but only high-quality leadership carried this effect, referencing those with medium-quality leadership; this did not hold true for low-quality leadership. A potential explanation is that high-quality leadership is important if people with diabetes have experienced support for increased sickness absence or health problems before they were diagnosed with diabetes, i.e. the opposite of the healthy worker effect [43]. More research is needed in this area because it is important to know how management could help employees with diabetes remain productive workers [9]. Alternatively, high-quality leadership may lead to over-commitment [44], which may again entail adverse health outcomes, such as diabetes.

There is scientific support that chronic stress—or, especially, lack of control—might cause diabetes among females. We thought that this cohort would provide insight into the strength of the stress-diabetes link [21]–[23], not least because this cohort has elsewhere identified that psychosocial demands and depression played a role in long-term sickness absenteeism [19]. However, our findings did not support this theory.

### Obesity and work

Our results suggest that the occupational setting affects diabetes indirectly through the influence of obesity and physical inactivity. Referring to diabetes as a ‘lifestyle disease’ is misleading, implying that there is something wrong with the character of the employee, when the truth is that what we call ‘lifestyle’ is a combination of structural conditions for health and capacities for coping with both living and working conditions. Based on the present study, the occupational setting may be more important for the prevention of diabetes than previously thought. Our survey confirms that occupational risk factors were associated with obesity to a much higher extent than was seen for diabetes.

Another challenge is that the causes of obesity are tightly interwoven with parallel factors from both working and non-working life. Traditionally, obesity has not been regarded as an occupational health and safety (OHS) issue, but as a matter for workplace health promotion (WHP). However, more than thirty years of WHP have not been able to reverse the rising prevalence of obesity, and initiatives addressing nutrition and physical activities have only showed modest effect [45]–[47]. Lifestyle is not simply a confounder to occupational risk factors; it is both a causal factor and mediator because work has become so much more sedentary [48]. One would have expected health care workers to be in relatively good physical shape because they walk, lift, move a lot and generally have physically demanding work [18], [20]. However, new research shows that the majority of people who are considered to have strenuous physical work may still not reach activity intensity levels that could improve physical capacity [49].

T2DM has hitherto been considered as a condition only affecting the elderly and caused by factors related to individuals' private lives; it has, therefore, viewed as largely irrelevant to OHS. This view will need to be revised as both obesity and diabetes become more prevalent within the working population. Even a small increase in diabetes prevalence will have considerable impact on the labour market. The gradually increasing pension age means that diabetes will occur in many more people while they are still active in the labour market [50]. Instead of continuing with WHP alone, the challenge will be to investigate if it is possible to organise work itself to support and foster health promotion. If so, the occupational arena may be able to contribute much more to the improvement of public health than is the case today.

### Strengths and limitations

A study limitation is that all baseline measures, including BMI, were based on self-report. However, self-report is expected to provide a reasonably reliable, albeit conservative, categorisation of obesity vs. non obesity [51]. A second limitation is that the DNDR does not distinguish between type 1 and type 2 diabetes; however, we estimate this had a minimal effect on our findings. Due to the low number of males in the survey, the results might not be generalizable to similar jobs with a different gender distribution. The dichotomous measure of ethnicity is probably too simple, since we know that Non-western populations have a higher diabetes risk than people of Western or Danish origin. However, the relatively low number of ethnic minority health workers hindered further stratification. One of the strengths of the study is the large sample size and high response rate. In addition, our ability to link high quality registers covering the entire Danish population with individually based information on work and lifestyle is unique. The homogenous population is also a strength of the study because it reduces the potential for confounding from the social gradient of health.

## Conclusions

In conclusion, diabetes is notably prevalent among health care workers. However, the risk of developing diabetes was mediated through obesity and physical inactivity, which play a causal role whether they are influenced by work or non-work lifestyle factors. Factors from the occupational setting were also associated with obesity. The importance of the occupational setting should therefore be reconsidered in the fight against diabetes. Obesity could be the target for interventions and diabetes would be a suitable disease model for surveillance of long-term effects. Finally, DNDR is a new and promising tool that needs to be explored further to elucidate its future value for monitoring large scale trends in the development of diabetes in the working population.
